# Successful management of scalp avulsion with full‐thickness graft

**DOI:** 10.1002/ccr3.6094

**Published:** 2022-07-25

**Authors:** Mohammad Badr Almoshantaf, Sarya Swed, Abdulkader Hajjar, Ala’a Alshawaf, Ahmad Nabhan, Bayan Zetani, Aladdin Etr

**Affiliations:** ^1^ Department of Neurosurgery Ibn Al‐Nafees Hospital Damascus Syria; ^2^ Faculty of Medicine Aleppo University Aleppo Syria; ^3^ Department of Plastic Surgery Aleppo Hospital Aleppo Syria

**Keywords:** accident, case report, graft, scalp avulsion

## Abstract

Scalp avulsion injuries are one of the life‐threatening traumatic injuries. Rapid management in the emergency department is vital to the successful rescue of an avulsed scalp. There are many replantation methods to treat scalp avulsion, so the best aesthetic and functional results are achieved. Skin grafting, free flaps, microvascular surgery, and hyperbaric oxygen are all suitable for reconstructive plans. We report a rare case of a scalp avulsion injury in a female due to an entanglement of a headscarf in a motorized machine resulting in defects and tissue loss of the hair‐bearing skin, nasal area, forehead, left ear, and bilateral eyelids and eyebrows. Preoperative management included early blood transfusion, intravenous fluids, and wound compression after rapid physical examination. Reconstructive surgery was performed using a full‐thickness skin graft and the outcomes were pristine. There are no clear guidelines to determine which reconstructive method is superior to another in each condition. Our case demonstrates that relatively primitive methods like skin grafting can give greater results if done with circumspection.

## 1 INTRODUCTION

The scalp is considered a mechanical barrier that prevents bruising and provides an important aesthetic appearance. Scalp ruptures are uncommon but serious. Severe traumatic injuries such as dog bites, high‐speed road collusions, or industrial accidents can damage hair‐bearing units and may result in an avulsion of the whole layers of the scalp.^1^

It is important to conduct a comprehensive examination of the body before starting treatment. Some injuries will be fatal if left untreated such as pleural effusion, pneumothorax, and intracranial hemorrhages. Hypovolemic shock may be seen in such injuries and must be detected and managed early by both blood and fluid administration.^2^

Avulsions of the scalp are painful and difficult to treat. The most successful and available therapeutic option is surgical replantation. However, failure of this process leads to undesirable results such as thermal wounds, infection, prolonged hospitalization period, and bad cosmetic outcomes.^3^

In this paper, we present a 10‐year‐old girl that had her scalp ruptured due to an electric bicycle accident. This avulsion extended to involve other structures. Such extensive injury with its management and outcome is a rare encounter in the literature.

## 2 CASE PRESENTATION

**FIGURE 1:**
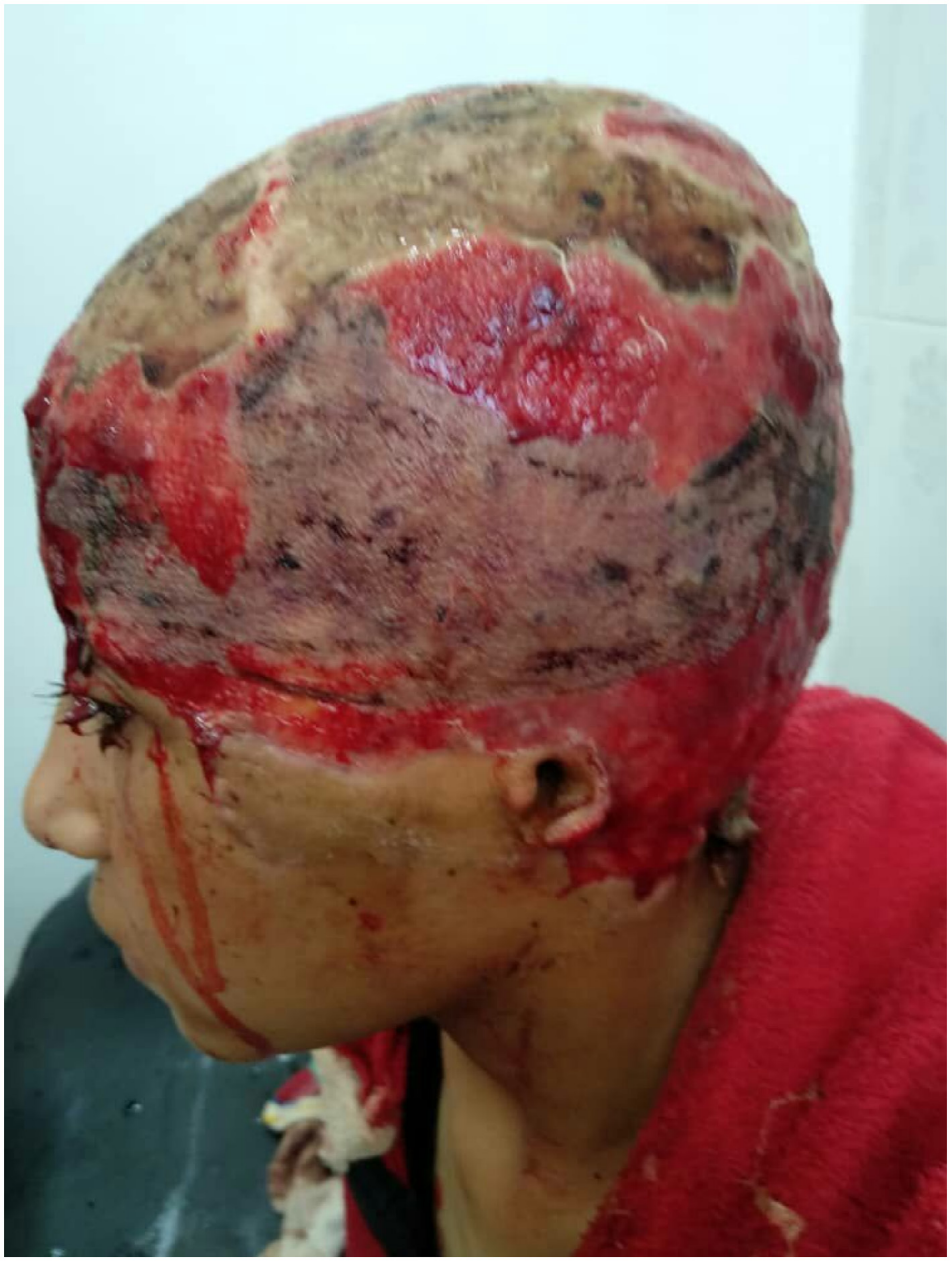
Scalp Avulsion injury on presentation

A 10‐year‐old girl presented to the emergency department with a complete scalp avulsion as a result of an electric bicycle injury. Initial observation (Figure 1) revealed that avulsed tissue extended to nose root, bilateral supra‐tarsal folds of the temporal region including eyebrows, and the left auricle. Additionally, the frontal bone was completely naked.

The patient on presentation was oriented and fully responsive with a Glasgow score of 15. There were no nausea, vomiting, or convulsions. The blood pressure was 100/50 and the heart rate was 106. She had no prior medical problems and was not on any medications at the time of her injury. On clinical examination, there were no fractures or bruises in her trunk or limbs. Initial management included intravenous fluids and antibiotics, blood transfusion, and topical anti‐septic.

Her laboratory investigations showed low levels of blood component; red blood cells were 1.84 × 10^6^/μl, hemoglobin was 5.6 g/dl, HCT was 16.1%, platelets were 103 × 10^3^/μl, and there was a slight elevation in granulocytes 80.9%. Other laboratory tests were normal.

**FIGURE 2:**
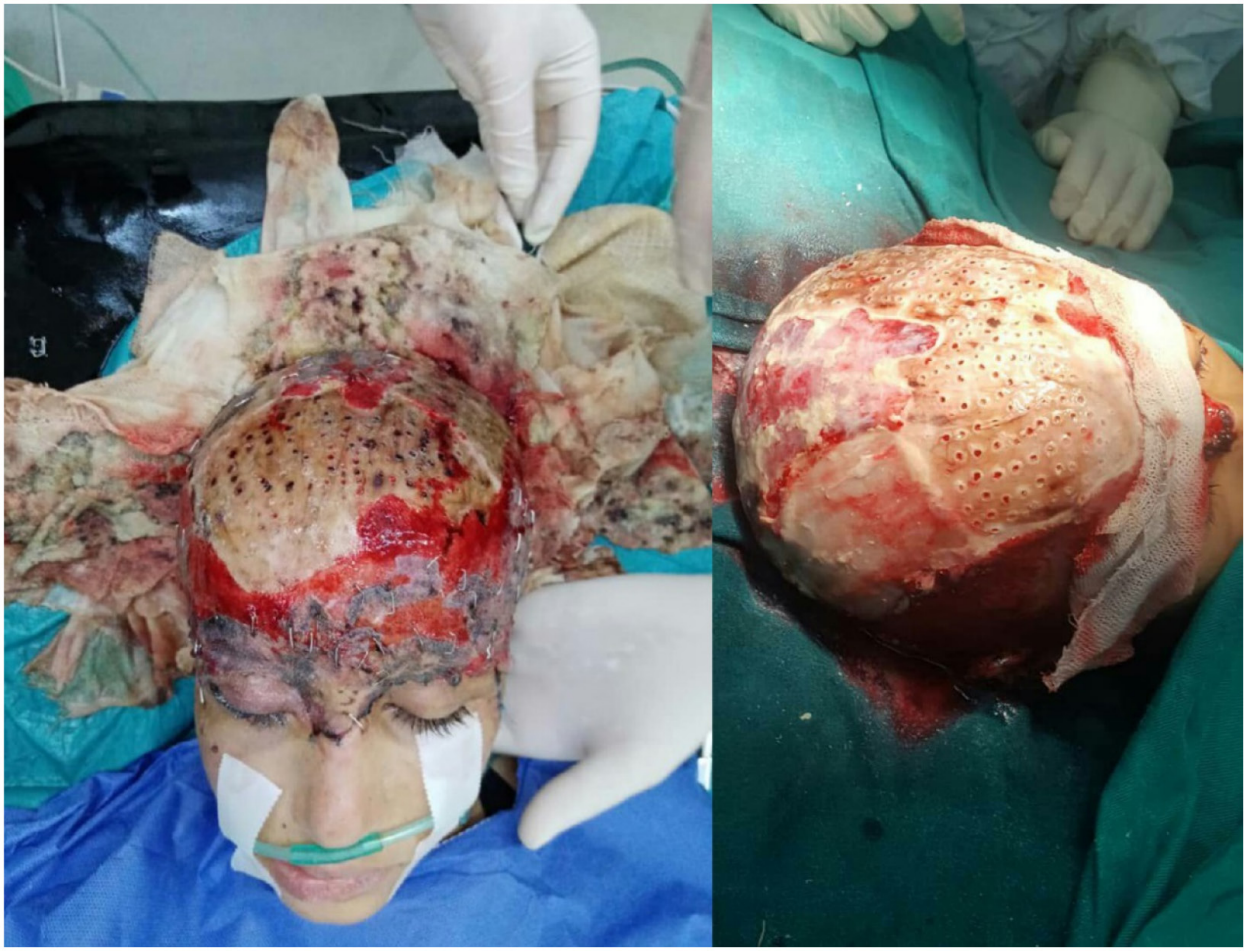
Reconstruction operation using full‐thickness graft from thigh

Soon after, we arranged a scalp reconstructive procedure in which we harvested a full‐thickness skin graft from the anterior right thigh and was expanded by a Zimmer dermatome mesher. Edges of the graft were attached to the scalp using staplers and nylon stitches (Figure 2).

**FIGURE 3:**
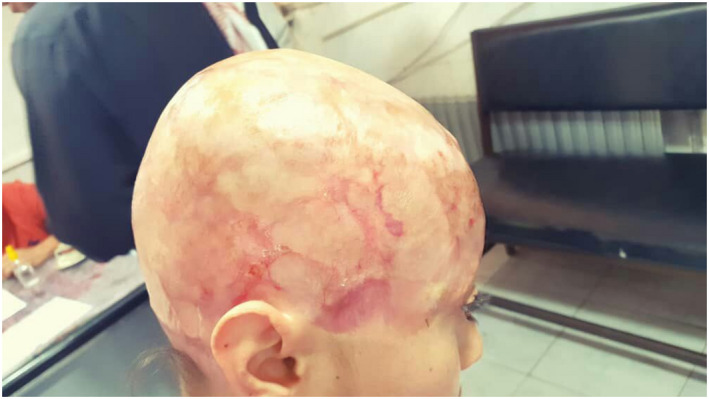
Outcome of reconstruction grafting

Several follow‐ups revealed healthy and uncompromised graft but with complete both hair and sensory loss (Figure 3). Finally, the patient was able to return to her normal life and she reports no difficulty in carrying out daily activities.

## 3 DISCUSSION

Total scalp avulsion is a serious injury because of its devastating effect on both the patient's general health and aesthetics. However, this type of injury is considered to be of a rare occurrence and could mostly be encountered during industrial or high‐speed road accidents. Our case had a scalp avulsion extending to the temporal and frontal bearing skin, the nasal bridge, and left ear's auricle due to an electric bike accident.

Most avulsed‐scalp‐related injuries are associated with hypovolemic shock and facial trauma. Despite that no signs of hypovolemic shock were clinically observed, prophylactic fluids administration was still ordered in emergency settings.

In most similar injuries, a sole or combined injury of blood vessels could be encountered. This includes superficial temporal artery, supraorbital artery, occipital artery, and facial artery. These are rich arterial and venous arcades in the subcutaneous layer above the epicranial aponeurosis.^4^, ^5^ Therefore, these injuries should be treated first to evade the possibility of developing any surgical contraindication. Fortunately, we found no similar injures as our case showed an acceptable patient's general condition. She was fully awake, self‐aware, and there were no signs of secondary injuries.

Usually, due to the extensive blood supply to the scalp, hemorrhaging of the scalp may appear profuse and should always raise suspicion of intracranial and cervical damage. Thus, patients should be examined thoroughly during the secondary survey by taking cervical spine and brain computed tomography to all patients in order to exclude surgical contraindications. As indicated in this case, test results proved no signs of cervical or intracranial damage.

Surgical replantation is the optimal treatment of the avulsed scalp that was first described by Miller et al. in 1976.^3^ A successful replantation can well restore the hair‐bearing aesthetic unit that is irreparable by other types of reconstruction.^6^ Many surgical techniques were used for managing these types of skin defects usually depending on the defect size including microvascular surgery, skin grafts, and free flap techniques. Free flap techniques allow for reliable wound closure while providing a variety of reconstructive options. The most common flaps that are used to cover the scalp are radial forearm flap, latissimus dorsi free flap, serratus anterior flap, and anterolateral thigh flap.^6^

Another method is presented by Khandelwal et al. (a successful use of hyperbaric oxygen therapy for a complete scalp degloving injury),^7^ discussed a case of a 43‐year‐old woman who presented with a complete scalp degloving injury, the result of a tractor‐powered take‐off that caught her hair, after initial evaluation and stabilization, the patient underwent a microsurgical replantation procedure after 4 h of her injury and lasted for 4 h, during the procedure, only the superficial temporal arteries were re‐anastomosed with no venous anastomosis possible due to the extent injury of the scalp, the surgeons then administered Hyperbaric oxygen (HBO2) treatment at 2.5 atmospheres absolute (ATA) for 90 minutes after surgery due to duskiness of the flap, further HBO2 treatments were administered and a post‐operative leech therapy for 10 days. At one‐year follow‐up, the area of granulation tissue was reduced, with no detectable hair growth or nerve function. Khandelwal et al. showed in their case that the use of (HBO2) therapy could increase the chances of tissue survival.^8^

The main reason we went for full‐thickness graft is the lack of proper equipment at our hospital. Microvascular surgery, free flaps, or HBO2 therapy were all out of reach. Despite that, grafting appears to have great results when certain indications are considered.

## AUTHORS CONTRIBUTIONS

All authors have read and approved the manuscript. MBA served as lead in writing and reviewing the manuscript. SS, AH, AA, AN, BZ, and AE contributed to writing the manuscript.

## CONFLICT OF INTEREST

No conflict of interest exists in the submission of this manuscript.

## ETHICS APPROVAL AND CONSENT TO PARTICIPATE

Ethical approval was given by the Aleppo University Hospital and the parent's patient has given their parental consent for this study.

## CONSENT

Written informed consent was obtained from the patient's parent for the publication of this case report and any accompanying images. A copy of the written consent is available for review by the Editor of this journal.

## Data Availability

All data generated or analyzed are included in this article.
